# Great Challenges Toward Sports Injury Prevention and Rehabilitation

**DOI:** 10.3389/fspor.2020.00080

**Published:** 2020-07-03

**Authors:** Pascal Edouard, Kevin R. Ford

**Affiliations:** ^1^Inter-university Laboratory of Human Movement Science (LIBM EA 7424), University of Lyon, University Jean Monnet, Saint Etienne, France; ^2^Faculty of Medicine, Department of Clinical and Exercise Physiology, Sports Medicine Unit, University Hospital of Saint-Etienne, Saint-Etienne, France; ^3^Department of Physical Therapy, Congdon School of Health Sciences, High Point University, High Point, NC, United States

**Keywords:** health protection, sports injury prevention, injury risk, sports rehabilitation, multidisciplinary approach, multifactorial approach

## Introduction

While the positive benefits of sport participation are numerous, unfortunately this is balanced by the negative effects of injury (Engebretsen and Bahr, 2005; Merkel, 2013; Malm et al., 2019). Sports injury prevalence and incidence vary according to sports and population. And unfortunately, short- and long-term negative consequences can impact a variety of different domains (e.g., physical, psychological, sport, professional, financial, or social). Whatever these impacts, there is a need to reduce the occurrence and consequences of sports injury in order to allow a healthy and sustainable sports participation (Engebretsen and Bahr, 2005). This is the important challenge of injury prevention and rehabilitation! This challenge includes primary prevention (i.e., to reduce the occurrence of the first injury event), secondary prevention (i.e., to reduce the occurrence of reinjury after a first injury event) and tertiary prevention (i.e., to reduce the occurrence of sequalae) (Commission on Chronic Illness, 1957).

The popular saying, “prevention is better than cure,” has been known for quite some time. While large efforts have launched to continue to engage in this challenging goal of injury prevention, it still remains a real problem in sports. This could be due to the complex and multifactorial nature of sports injury (Meeuwisse, 1994; Bittencourt et al., 2016; Pol et al., 2019), which makes its “prevention” / “reduction” difficult. It seems that sports injury is not the result of one unique cause but likely the combination and interactions of several factors (including among others intrinsic and extrinsic risk factors and injury mechanism) (Meeuwisse, 1994; Bahr and Krosshaug, 2005; Bittencourt et al., 2016).

In order to face this challenging problem of sports injury, there is thus even more of a need to understand sports injury: How to monitor sports injury? What are the risk factors? How these factors interact? What is the healing process injured tissue? How can we optimize the process of healing, functional recovery, and safe return to sports? Then, there is a need to continue to reflect and develop strategies that can help to reduce the occurrence and recurrence of sports injuries: How can we play on/modify these factors to reduce the occurrence and/or recurrence of sports injuries? Which strategy or combination of strategies can reduce the occurrence and/or recurrence of sports injuries? Are these strategies efficient to reduce the occurrence and/or recurrence of sports injuries, in the context of scientific studies and in real life? How can we implement these strategies? How can athletes be compliant with these strategies? To answer these questions and reach this great challenge of injury prevention and rehabilitation, we believe that there is not one way, we believe that approaches should be comprehensive, multidisciplinary and holistic, including contributors from different fields, with communication between them and by embracing new fields.

## Prevent or Reduce?

Before going to concrete aspects, there is maybe a need to improve knowledge and accuracy on some conceptual and terminological aspects.

“Prevention” is a widely used term, however, this is likely not the most appropriate or feasible term within sports. This term is well-known and recognized as a banner of work which aims to protect the health of athletes, especially injuries and illnesses, perhaps thanks to the important efforts of the Oslo Sports Trauma Research Center and the IOC toward injury and illness prevention (Engebretsen and Bahr, 2005; Ljungqvist, 2008; Engebretsen et al., 2014). Although this term could be useful to describe the field (as we use for the name of our section *Injury Prevention and Rehabilitation*), it is maybe not the most appropriate when we want to accurately discuss about the concrete goals. Indeed, “prevention” means no occurrence of injuries, which is probably not possible. So, most appropriate terms would probably be “injury control” or “injury risk management” or “injury risk reduction” (Avery, 1995; Webster and Hewett, 2018). Other terms are also used on our field that deserve to have clear definitions for proper use, such as for instance “efficacy,” “effectiveness,” “compliance,” “prediction,” “prognostic.” Therefore, we believe that some discussions, researches and/or consensus should clarify these aspects.

In addition, sports injury prevention research is often modeled around the classic four-step sequence presented by van Mechelen et al. (1992) nearly 30 years ago. This model has provided a conceptual framework to monitor progress and effectiveness of decreasing the incidence of a variety of sport injuries (Edouard et al., 2015; Hewett et al., 2016). The “sequence of prevention” conceptual framework was extended in 2006 by Finch (2006) to phases related to the implementation of prevention measures and evaluation of real-world impact. Recently, Bolling et al. (2018) revised the four-steps sequence by improving the first step of the sequence extended to exploration of the context of the sports injury. Other frameworks have been developed to detail some steps of the sequence or some specific aspects, for instance, concept of sports injury (Timpka et al., 2014b), etiology of sports injury (Meeuwisse, 1994), understanding injury mechanisms (Bahr and Krosshaug, 2005), a biomechanics-focused model (Hewett and Bates, 2017), complex systems approach (Bittencourt et al., 2016; Pol et al., 2019), risk factor-based categorization of the prevention (Jacobsson and Timpka, 2015), prevention measure implementation (Tee et al., 2020), and individualized approach (Roe et al., 2017). These conceptual frameworks of sports injury prevention research can continue to benefit from improvements or details to help researchers and/or practitioners.

## Primary and Secondary Prevention = Same Fight!

Methodology used in primary prevention could seamlessly assist secondary prevention and vice versa (Hewett and Bates, 2017; Cools et al., 2020). In addition, given the high prevalence of sports injury, a large percentage of athletes will participate in sport with history of previous injury. Therefore, the need for secondary prevention is ongoing and increasingly more important as athletes age. However, primary and secondary approaches are sometimes compartmentalized; sports scientists and coaches may be more involved with primary prevention, while health professionals involved with secondary prevention. Consequently, scientific literature may also be compartmentalized. Therefore, we strongly support that all knowledge regarding both primary and secondary prevention should be directly translated to all stakeholders (applied, clinical). In addition, we suggest increased communication and collaboration between professionals and community to reach success in this challenge.

## Specificity of Sports Rehabilitation

Secondary prevention can be addressed through rehabilitation (Hewett and Bates, 2017; Cools et al., 2020). This particular phase of the sports injury management has some specificities. It aims to orient/guide the injured tissue healing process, restore the function, and help the patient/athlete return to sporting activities while at the same time minimizing the risk of reinjury. This multi-goal management is currently approached mainly through biological/physical aspects (e.g., physiological, biomechanical…). However, psychological, social and contextual factors play a critical role in the recovery of patients/athletes after sports injury, and should be taken into account in this phase of the sports injury management.

Sports rehabilitation should thus be done in a multifactorial biopsychosocial approach, bringing the patient/athlete from injury to return to his desired activity, by taking into account the consequences of the sports injury at these different levels (Ardern et al., 2016; Van Melick et al., 2016; Cools et al., 2020).

## Unity is Strength: Need of Multidisciplinary Teamwork!

To face the problem of sports injury, everyone is needed! Each person has a different experience, expertise, and view of the problem. So, it is important to encourage and act on the input from all parties involved. This implies a multidisciplinary approach, with inputs from several fields (e.g., sports medicine, sports and exercise science, physical conditioning and training, biomechanics, nutrition, physiology, psychology, sociology, data science…). This implies for instance at a field level that health professionals and coaching staffs, who are facing the same problem of sports injury, share their points of view, arguments, proposals of management in order to find the optimal solution for athletes. Likewise, this should be extended to other fields working with athletes in order to create a cohesive multidisciplinary team. This approach should be favored at the field/clinical and research levels.

Such an approach implies communication to go beyond discussions simply within a field and extend to discussions between diverse fields of interest. This also means for athletes' monitoring or research purpose collecting data from different fields, and probably makes choice or compromise given the amount of data this can represent. These discussions or choices are probably not easy because of some conceptual or language barriers, potential for competition, or perceived skepticism. There will maybe a need to structure discussion / choice, and there is a need to clarify the responsibility of each other, especially when coming the decision. But we believe that this is a relevant orientation to overcome the great challenge of sports injury prevention and rehabilitation. We suggest this would be a win-win approach for all stakeholders. The resulting benefits of discussion, exchange, and collaboration would be greater than the sum of each individual input.

## Need for a Holistic and Individual Approach

Recent evidence supports that several factors of varying types can play a role in the occurrence of injury or reinjury (Kerkhoffs et al., 2012; Hewett et al., 2016; Green et al., 2020). In addition, each athlete will respond differently to these factors and combination of factors; each will not have an injury for the same reasons. Hence, patients will respond differently to the injury and its consequences. This is supported in contemporary sports injury management which should utilize a bio-psycho-social approach at an individual level and grounded in evidenced-based practice. Thus, efforts should be made in sports injury research to provide knowledge and evidence in each of these different fields, and if possible, combining all these fields.

Given the complexity of sports injury, the research approach currently simplifies the problem, but there will need to go deeper in complex multifactorial individualized approach to better meet the “reality” of sports injury (Meeuwisse, 1994; Bittencourt et al., 2016; Pol et al., 2019).

There is thus a need for a more complex approach, a comprehensive holistic and individual approach, as for understanding the determinants of the sports injury as for the development of strategies that aim to reduce the occurrence of injury or reinjury. Examples are proposed through conceptual or perspective articles (Mendiguchia and Brughelli, 2011; Mendiguchia et al., 2017; Buckthorpe et al., 2019), and there is now a need to provide supporting evidence of the theses approaches.

## Improve Methodological and Analytical Approaches

One of the challenges in injury prevention research is to capture the outcome, i.e., sports injury. Efforts have been done to develop and improve methodology for recording and reporting injuries (Hagglund et al., 2005; Fuller et al., 2006; Junge et al., 2008; Timpka et al., 2014a; Bahr et al., 2020) and should continue to most accurately capture injuries and their complexities.

Alternative analytical approaches of effectiveness of injury prevention measures can use as outcome the consequences of the sports injury at physical, psychological, social or financial levels. Injury prevention is of course useful to reduce the occurrence or reoccurrence of injuries, but also that of sequelae (Engebretsen and Bahr, 2005) or of the financial impact (Krist et al., 2013). Taking into account the economical burden of sports injuries (Hickey et al., 2014; Hespanhol Junior et al., 2017) could also be a way to improve stakeholders adherence to prevention and increase means for sports injury prevention and rehabilitation at the practical and research levels.

The multifactorial biopsychosocial approach leads to the need of adding in the measurements, data collection or monitoring, information related to the sports injury and the injured athletes, taking into account their multifactorial and complex nature, as well as about the context including individual, socio-cultural and environmental/policy levels (Bolling et al., 2018).

The multifactorial approach leads to multimodal methodological approaches. Traditionally quantitative analyses are used in sports injury prevention and rehabilitation research. There is thus a need to improve knowledge through qualitative approach (Bolling et al., 2018, 2019a,b). There is also a need for more behavioral approach when it comes to actual sports injury prevention (Verhagen et al., 2010) and when we aim increase compliance to prevention measures.

The multifactorial approach leads to analytical challenges. Indeed, this implies increasing the magnitude and type of data, which is of interest to fit the complex nature of sports injury, but can be difficult to managed by traditional analytical approaches, and for sure imply the collaboration with statistical and data science community (Casals and Finch, 2018; Nielsen et al., 2020b). To analyse complex interactions between factors and/or between sports injury and factors, there is a need for new analytical advances (Bittencourt et al., 2016; Nielsen et al., 2020b). As a consequence, other fields of data analyses, such as for instance machine learning, will continually be embraced in the future (Bittencourt et al., 2016; Ruddy et al., 2019). These analytical approaches may help analyse complex interactions as well as estimating the risk of sports injury occurrence, with application to understand the sports injury as well as to reduce their occurrence or recurrence (Bittencourt et al., 2016; Ruddy et al., 2019).

In addition, there is a need to use appropriate methodologies to analyse the efficacy of each of these strategies. Randomized Controlled Trial is currently the gold standard to analyse the efficacy of an intervention (Philipps et al., 2009), it is the design that should allow the highest level of evidence by minimizing the risk of bias. However, such design may not be the most relevant to reflect the reality of sports given, among others, the risk of low compliance (Nielsen et al., 2020a). We could benefit from improvement in methodological design inspired from other research fields. In addition, usual analytical approaches, such as *intention-to-treat, per protocol or as treated* analyses, can lead to bias, especially in the context of low compliance (Edouard et al., in revision). Therefore, there is a need to explore other analytical approaches, as IV analysis, or other G-estimation, which can address some of the problems that arise from low compliance without losing the value of randomization and can also be helpful in observational studies (Edouard et al., in revision).

## Conclusions

Although injury prevention and rehabilitation are not new disciplines, there is still an unmet need to improve knowledge toward theoretical understanding on epidemiology, risk factors, and injury mechanisms, as well as on practical strategies that can reduce the risk of sports injury or reinjury and of sequalae after injuries. Given the complex nature of injury, a holistic multifactorial biopsychosocial approach is needed through comprehensive, multidisciplinary and individualized approach to reach this great challenge. We therefore hope that this new section *Injury Prevention and Rehabilitation* of the *Frontiers in Sports and Active Living* can contribute to this improvement of knowledge, but also positively impact the sustainable and safe participation and short and long-term health of athletes.

## Author Contributions

All authors listed have made a substantial, direct and intellectual contribution to the work, and approved it for publication.

## Conflict of Interest

The authors declare that the research was conducted in the absence of any commercial or financial relationships that could be construed as a potential conflict of interest.
